# Enhancing Trauma Patient Experience Through Education and Engagement: Development of a Mobile Application

**DOI:** 10.5435/JAAOSGlobal-D-20-00025

**Published:** 2020-03-17

**Authors:** Benjamin R. Childs, Mary A. Breslin, Brendan A. Andres, Anna Swetz, Sarah B. Hendrickson, Timothy A. Moore, Vanessa Ho, Heather A. Vallier

**Affiliations:** From the MetroHealth Medical Center, Case Western Reserve University, Cleveland, OH.

## Abstract

The purpose was to determine the utility of an open access mobile device application (App: http://bit.ly/traumaapp) to improve patient education and engagement.

**Methods:**

A patient education app was developed with information regarding injury, treatment, and recovery for orthopaedic and other injuries. Data regarding usage, satisfaction, and desired improvements were gathered.

**Results:**

The app was downloaded 725 times, and the pages in the app were viewed 9,043 times in 34 months. User sessions >2 minutes accounted for 34%. Participation was less in those older than 55 years (12% versus 68% *P* < 0.001). Sixteen percent of patients did not have a device to use the app. Most (55%) rated it as helpful or extremely helpful; 78% of users were likely to recommend it. Patients most frequently suggested more information on other injuries and simpler language.

**Discussion:**

There was strong interest in this simple, free patient education app. Despite an urban, trauma population, five of six patients had access to a device that could load the app. Nearly half of the patients downloaded an orthopaedic patient education app when offered. Those who did not use the app were more likely to be older than 55 years. This represents an innovative opportunity for education and engagement of our patients and their families.

In 2016, the American Orthopaedic Association joined the American Medical Association, the Institute of Medicine, and the Agency for Healthcare Research and Quality in acknowledging health literacy as a critical issue for improving patient outcomes. Low health literacy is associated with poor outcomes for orthopaedic conditions.^1-3^ Furthermore, low health literacy increases healthcare costs by up to four-fold.^4^

Musculoskeletal health literacy is consistently lower than health literacy for other medical conditions. Although 48% of patients do not have high enough health literacy to understand their discharge instructions, in orthopaedics, that number is estimated at 68%.^5^ However, this may be an overestimate for the orthopaedic trauma population. These data are mitigated by the more affluent and educated outpatient elective orthopaedic population. It has been shown that orthopaedic patients who present to the emergency department (ED) have lower health literacy than those who present to outpatient clinics.^6^ This corroborates evidence that orthopaedic trauma patients have difficulty understanding their injury, treatment, and postoperative instructions.^7^ However, gaps in understanding are not permanent; it is possible to increase health literacy with simple interventions.

In orthopaedic trauma patients, a well-designed combination of images and text increased patients' understanding of treatment and discharge instructions after open reduction internal fixation.^8^ Furthermore, technology has been shown to be beneficial in the delivery of information.^9^ Patient comprehension of informed consent increased with the addition of a simple and informative website to the consent process.^10^ Mobile applications (apps) have the potential to augment existing literacy interventions; however there are few apps available for patient education.^11^

We describe the development of simple patient education materials. The materials use short sentences, common words, conversational tone, and relevant pictures to deliver content at a sixth grade reading level. We deployed these materials through an app. We hypothesized that patients desire more information, and they usually possess electronic devices with app capabilities. We further hypothesize that patients will choose to engage with an educational app when recommended by a healthcare provider. We report initial experience and feedback from trauma patients.

## Methods

The content for this app was developed by fellowship-trained orthopaedic, trauma, and spine surgeons with over 15 years of experience at a level 1 trauma center. The text was created using common words, short sentences, and conversational tone so that it can be easily read. Images that complemented the text were included with relevant explanations. The scope of the content was intended to cover most common orthopaedic trauma injuries. The topics cover upper and lower extremity, pelvic, and spinal injuries. Content for each injury included background, nonsurgical management, surgical management, and recovery.

The developed content was reviewed by multiple readers at different education levels to give diverse perspectives on simplification for the concepts. After development, the reading level was calculated and the content was refined (Table 1). Additional content relevant to orthopaedic trauma patients was indexed from public websites related to hospital offerings. This included information on providers and information regarding Trauma Survivors Network support services. The content was organized into an app released on January 3, 2017, on both Google Play and Apple App Store (http://bit.ly/traumaapp). Physicians, residents, and nurses at the founding site were made aware of the app. Posters were hung and advertisements were given out on the inpatient hospital floors and in the outpatient clinic, so as to encourage any trauma patients and their family members and friends to use the app. This was not structured or enforced, and the app usage was allowed to wax and wane organically. Staff usage, resident usage, and nurse usage were collected anecdotally.

**Table 1:**
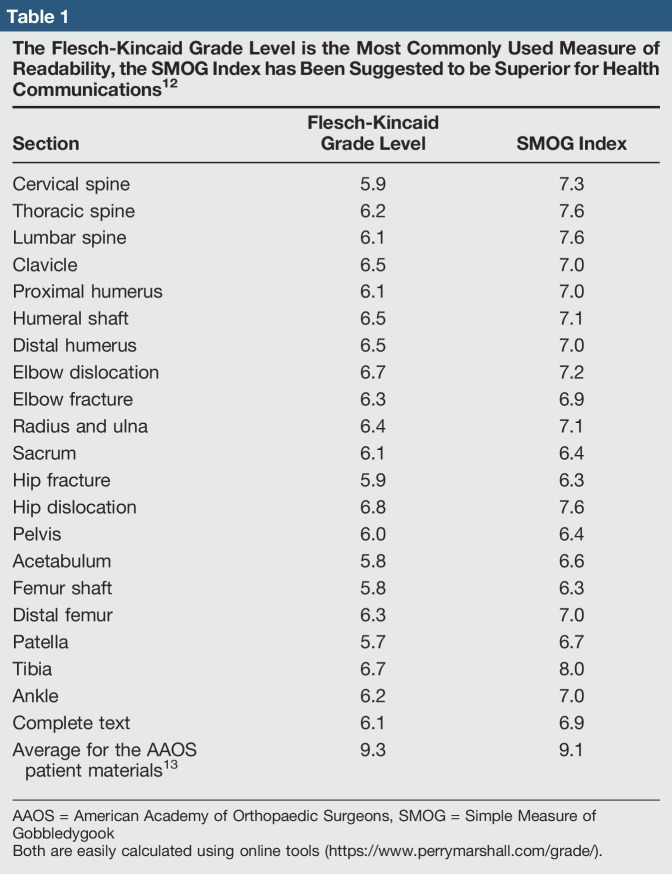
The Flesch-Kincaid Grade Level is the Most Commonly Used Measure of Readability, the SMOG Index has Been Suggested to be Superior for Health Communications^12^

Section	Flesch-Kincaid Grade Level	SMOG Index
Cervical spine	5.9	7.3
Thoracic spine	6.2	7.6
Lumbar spine	6.1	7.6
Clavicle	6.5	7.0
Proximal humerus	6.1	7.0
Humeral shaft	6.5	7.1
Distal humerus	6.5	7.0
Elbow dislocation	6.7	7.2
Elbow fracture	6.3	6.9
Radius and ulna	6.4	7.1
Sacrum	6.1	6.4
Hip fracture	5.9	6.3
Hip dislocation	6.8	7.6
Pelvis	6.0	6.4
Acetabulum	5.8	6.6
Femur shaft	5.8	6.3
Distal femur	6.3	7.0
Patella	5.7	6.7
Tibia	6.7	8.0
Ankle	6.2	7.0
Complete text	6.1	6.9
Average for the AAOS patient materials^13^	9.3	9.1

AAOS = American Academy of Orthopaedic Surgeons, SMOG = Simple Measure of Gobbledygook

Both are easily calculated using online tools (https://www.perrymarshall.com/grade/).

**Figure 1:**
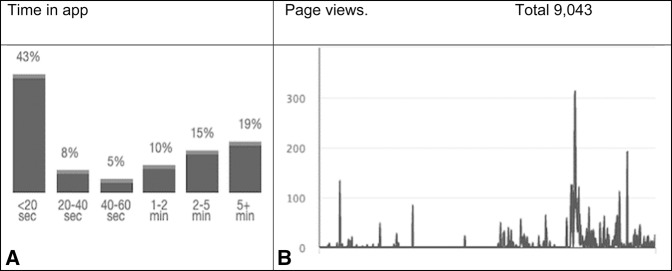
Chart showing time spent in app (per session) and **B**. Total page views **A**. Greater than one-third of the sessions were longer than 2 minutes. **B**, Variability in usage, as shown by number of page views, with occasional high-frequency users.

Data were collected through the app and in a patient survey. Data from the app stores included downloads, location, frequently used features, page views, and time in app. The outcome “Downloads” was defined as the number of app downloads per time period. The time period was adjustable by days, weeks, or months. Location was defined using a map provided by the app platform that showed the location with granularity to the level of a city. Frequently used features were defined as the pages in the app that were used most frequently and was cumulative over the selected time frame. Page views were defined as unique visits to each section of the app and were cumulative over the selected time frame. Time spent in app was defined as the duration in minutes of each session or time a user opened the app. Time was recorded in 20 second blocks for sessions below one minute and then 1 to 2 minutes, 2 to 5 minutes, and greater than 5 minutes (Figure 1). The ratio of views to downloads and usage was collected from the app platform.

Patients and caregivers were offered a brief survey regarding their experience with the app 24 hours after exposure (Supplemental content, http://links.lww.com/JG9/A70). Surveys were administered by a trained researcher not involved in the care of the patients. Age, relationship to patient, functionality, and desired improvements were gathered from the survey.

## Results

Data in this study come from two sources: app analytics and surveys. First, the results of the app analytics are as follows. From the initial publication to this analysis (January 3, 2017, to October 10, 2019), the ratio of search result appearances, to product page views, to downloads was approximately 350:7:6. In the Apple App Store, 532 of the 598 product page views resulted in downloads (89.8%). In total, the app was downloaded 725 times from Google Play and Apple App Store. Of these 725 downloads, 504 (70%) were in the metropolitan regions of the level 1 trauma centers where this study was conducted.

User sessions (individual uses of the app) were split in a bimodal distribution between those who used the app for more than 2 minutes (34%) and those who used it for less than a few seconds (43%). Overall, the pages in the app were viewed 9,043 times in 34 months with variable spikes in usage during single days. Patients visited “Your Injury,” “recovery timeline,” and “FAQ (frequently asked questions)” most often.

**Figure 2:**
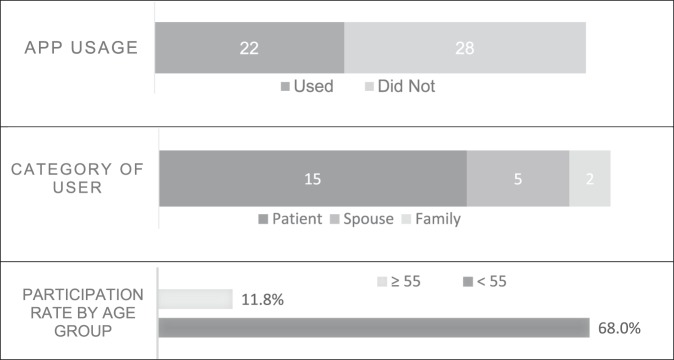
Chart showing patient and caregiver surveys collected during the injury admission.

The following results are from patient and caregiver surveys (Figure 2). Patient and caregiver surveys were collected from 50 patients who had been offered the app as part of their care. In total, 22 of 50 patients (44%) used the app. Of the people surveyed who did use the app, 68% were patients, whereas 23% were spouses and 9% were family. The survey results showed no difference in app participation between men and women (both 48%). Participation was less in those aged 55 or older (12% versus 68%, *P* < 0.001). Differences in participation were also reflected in the mechanism of injury with 82% of those in motor vehicle collision (MVC) versus 9.1% of those who fell from standing deciding to use the app, *P* < 0.001. Of the 28 survey responders who did not use the app, 8 (29%) responded that they were “not interested” in the app and another 8 (29%) responded that they did not use the app because of having no phone and 3 (11%) declined because of having a first language other than English. Anecdotally, patients without phones frequently had them damaged in the event that led to the trauma. Seventy-eight percentage of those who used the app were likely or very likely to recommend it. Of the patients who used the app, all reported that it greatly helped their ability to understand their injuries (mean 4.2 on 5 point scale). When asked for improvements to the app, patients suggested more information on other injuries most often, accounting for 25% of suggestions.

## Discussion

Low health literacy is prevalent among orthopaedic trauma patients. Improving orthopaedic literacy is a critical issue for improving patient outcomes.^1^ Owing to the combination of low literacy and lack of appropriate materials, one study found that only 45% of orthopaedic trauma patients were able to correctly identify their weight bearing status and a quarter did not know their deep venous thrombosis (DVT) prophylaxis.^7^ There is a clear need for appropriate patient education materials to improve the understanding of postoperative care.

Although it has been shown that well-designed materials can increase patient engagement, the difficulty of developing effective patient education materials is exemplified by the patient education materials on the American Academy of Orthopaedic Surgeons (AAOS) and the Pediatric Orthopaedic Society of North America websites. These materials are above the reading level of many patients.^14^ Although analysis showed that the mean reading level of AAOS articles has decreased from 2008 to 2016, 84% of patient education materials on the AAOS website are still written above the reading level of our patients.^13^ Detailed strategies for increasing the readability of patient education materials exist and include use of shorter sentences, common words, a conversational tone, and pictures with relevant text.^15^

We found that it is possible to develop patient education content at the appropriate reading level. With little cost, a free app was developed that contains educational content covering the breadth of orthopaedic trauma and related injuries. The material was written by experienced orthopaedic trauma and spine surgeons and general surgeons. The content was reviewed by a wide variety of stakeholders. The material in each section is at the sixth grade reading level and has relevant images. For each injury, there are a background, nonoperative, operative, and recovery sections. The information provides answers for patients but does not promise specific treatment. It is in line with the simple treatment principles and can be used at any facility. The app format allows for continual improvement. The material was expanded based on patient feedback to include general trauma injuries. The material will continue to be revised in the same fashion as our experience grows.

Patients consistently downloaded the app over the course of the study. This is evidenced by the large proportion of downloads that took place in the metropolitan areas where the study was conducted (70%). The unusually high rate of downloads per page view in the App Store (90%) also suggest people who found the app were looking for it. It is likely that most were referred by their providers. Furthermore, once the app was downloaded, it was viewed. Over one-third of the time patients opened the app, they spent more than 2 minutes interacting with it.

Survey data were necessary to investigate characteristics about users. We were able to supplement the data from app analytics with surveys from patients and caregivers. Survey data revealed that the app was not only used by patients but also frequently by spouses and family members. As expected, participation was greater among younger patients. We assume this is due to comfort with technology. This bias to younger patients was reflected in use by mechanism of injury with high energy mechanisms having more app usage than patients who fell from standing.

Survey data revealed that although lack of interest was given as a reason for not using the app, it did not account for most of the reasons patients did not use the app. Patients also cited not having a phone and lack of English language skills as major reasons they did not use it. There was concern that patients would not have devices capable of loading the app. However, only 16% of patients overall reported not having a capable device. Many of those who did not have a phone wrote in the survey that it was destroyed or otherwise lost in the event that caused their injury.

There are many limitations to this study. We did not attempt to associate app usage with clinical outcomes. We did not test patient comprehension after the use of the app. Therefore, we cannot show that these education materials benefited the patients. We surveyed only a portion of patients who were offered the app. The surveyed patients may have a selection bias and may not represent most patients. During the next stage of the development of this patient education app, we began systematic usage at three other level 1 trauma centers. We will determine if it is applicable in centers with different populations and variations in routine care. We also acknowledge the need for a robust strategy to communicate to patients and providers about the app, which we think will increase the usage. In future research, we aim to measure the impact of app usage on patient comprehension of discharge information.

In conclusion, we were able to create an app written at the sixth grade reading level with relevant images. The app was downloaded and used by patients. Usage information from survey data were consistent with app analytics. We think that there is a substantial opportunity for the orthopaedic trauma community to take a leading role in improving patient education and engagement.
